# Shape Effect on the Refractive Index Sensitivity at Localized Surface Plasmon Resonance Inflection Points of Single Gold Nanocubes with Vertices

**DOI:** 10.1038/s41598-019-50032-3

**Published:** 2019-09-20

**Authors:** Hui Bin Jeon, Philippe Vuka Tsalu, Ji Won Ha

**Affiliations:** Advanced Nano-Bio-Imaging and Spectroscopy Laboratory, Department of Chemistry, University of Ulsan, 93 Daehak-Ro, Nam-Gu, Ulsan 44610 South Korea

**Keywords:** Analytical chemistry, Photochemistry, Physical chemistry

## Abstract

Plasmonic gold nanoparticles with sharp tips and vertices, such as gold bipyramids (AuBPs) and gold nanocubes (AuNCs), have been widely used for high-sensitivity localized surface plasmon resonance (LSPR) sensing. However, conventional LSPR sensors based on frequency shifts have a major disadvantage: the asymmetry and broadening of LSPR peaks because of instrumental, environmental, and chemical noises that limit the precise determination of shift positions. Herein, we demonstrated an alternative method to improve the efficiency of the sensors by focusing on homogeneous LSPR scattering inflection points (IFs) of single gold nanoparticles with a single resonant mode. In addition, we investigated the effect of the shape and vertices of AuNCs on the refractive index (RI) sensitivity of homogeneous LSPR IFs by comparing with gold nanospheres (AuNSs) of similar size. The results show that for both AuNCs and AuNSs, tracking homogeneous LSPR IFs allows for higher RI sensitivity than tracking the frequency shifts of the LSPR peaks. Furthermore, single AuNCs with vertices exhibited higher RI sensitivity than single AuNSs of similar size in the homogeneous LSPR IFs. Therefore, we provided a deeper insight into the RI sensitivity of homogeneous LSPR IFs of AuNCs with vertices for their use in LSPR-based biosensors.

## Introduction

Plasmonic gold nanoparticles (AuNPs) have unique optical properties that depend on their shapes and sizes, and on the refractive index (RI) of the surrounding media. These properties are induced by the localized surface plasmon resonance (LSPR) effect^1–4^. When gold nanoparticles are irradiated, the conduction electrons on their surfaces are excited and collectively oscillate with the incident electromagnetic field. Furthermore, the strong interaction between gold nanoparticles causes light to be confined into sub-diffraction volumes^5,6^.

For many years, the optical properties of single AuNPs have been intensively investigated by far-field single particle imaging and spectroscopic techniques, such as scattering-based dark-field microscopy^7^ and absorption-based photothermal imaging^8,9^, without ensemble averaging. It has been reported that the LSPR of AuNPs is strongly dependent on the three-dimensional (3D) structure and size of the nanoparticles^2,10^, as well as on the RI of the surrounding medium^11^. Accordingly, by controlling these parameters, it is possible to tune the characteristic plasmonic properties for specific purposes and applications^12,13^. Furthermore, the AuNPs have unique intrinsic properties^11^, such as biocompatibility^14^, high chemical stability^15^, convenient surface modification with organic and biological molecules^16,17^, etc. The many advantages of AuNPs has thus led to their use in LSPR-based biosensors^18,19^. The conventional LSPR biosensors are based on AuNPs functionalized with receptors that confer specific binding abilities for target molecules, then the LSPR peak is shifted and dampened upon the attachment of the target molecules on the nanoparticle surface^20^. Thus, the LSPR changes of AuNPs is monitored by the shift of the peak maximum as well as broadening of the peaks^21^; such changes indicate the presence of target molecules^22^.

Despite the remarkable advantages of LSPR-based biosensors, they still have many fundamental limitations. First, the efficiency of LSPR-based sensor using AuNPs is low in comparison with surface plasmon polariton (SPP) sensors^23^. The accurate determination of LSPR properties is affected by a realistic representation of the wavelength-dependent dielectric function of the nanoparticles^10^. Therefore, simplistic models negatively impact the fundamental quantities that are necessary for the reliable fabrication of plasmonic devices^24^. Second, LSPR biosensors are limited by the unsymmetrical broadening of LSPR peaks when measuring the changes in the local environment at the nanoparticle surface^25^. It should also be noted that alterations in the shape of the LSPR peak can have a negative effect on the sensing efficiency^26^.

To overcome these limitations, recent studies have, for example, improved the effectiveness by using lithographic methods, but there are some disadvantages such as the high processing cost and low yield^23^. Recently, Chen and co-workers reported a different approach that evaluates the changes in LSPR curvature of ensemble samples with respect to RI changes^23^. They showed that higher RI sensitivity was obtained in the inflection points (IFs) located at the long wavelength side (or low energy side) of the LSPR extinction peak^25^. However, that report was based on only ensemble samples of Au nanoparticles rather than single nanoparticles. Very recently, a single particle study on homogeneous LSPR IFs of single Au bipyramids was reported, however, our understanding of the effect of the NPs shape on the RI sensitivity at LSPR IFs of single Au nanoparticles is still scarce^27^.

In this study, we carried out single particle studies to evaluate the shape-dependent RI sensitivity at LSPR IFs of homogeneous scattering spectra experimentally measured for gold nanospheres (AuNSs) and gold nanocubes (AuNCs), to compare structures with and without vertices. We investigated the LSPR sensing effect of single AuNSs and AuNCs deposited on a glass slide with three different surrounding media of known RI values (air, water, and oil). The results indicate that tracking the homogeneous LSPR IFs of AuNCs with vertices can be effectively used to develop LSPR-based biosensors with high RI sensitivity.

## Results and Discussion

### Characterization of AuNSs and AuNCs with vertices

**Figure 1:**
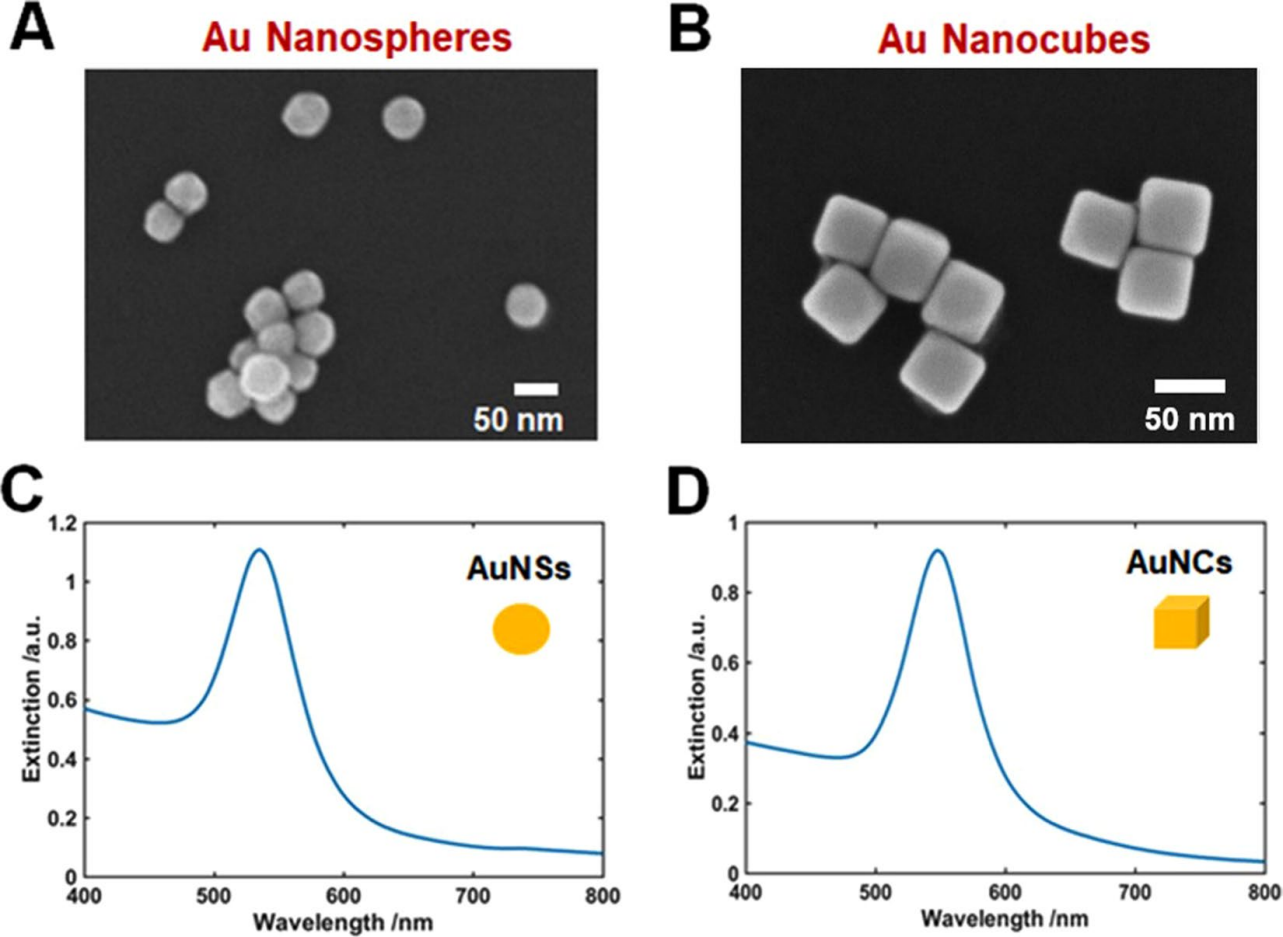
SEM images of AuNSs (**A**) and AuNCs with vertices (**B**). UV-Vis extinction spectrum of AuNSs (**C**) and AuNCs (**D**) dispersed in water.

The size and shape of AuNSs and AuNCs was characterized by SEM. Figure 1A,B show the SEM images of AuNSs (A) and AuNCs with vertices (B), with average sizes of 50.3 (±1.7) nm and 51.1 (±2.1) nm, respectively (Fig. S1). The size of 51.1 nm in AuNC indicates the length of one side of cube. The extinction spectra of both AuNSs (Fig. 1C) and AuNCs (Fig. 1D) was then obtained with a Varian Cary 300 UV-Vis spectrophotometer. We found that the extinction spectra obtained from AuNSs and AuNCs were very similar. However, the LSPR peak was seen at around 535 nm for AuNSs, while the LSPR peak was observed at approximately 547 nm for AuNCs dispersed in water. Furthermore, the LSPR linewidth was different for the AuNSs and AuNCs. In Fig. 1, the measurements at the ensemble level are limited by heterogeneity issues and, hence, single particle measurements are required for a better understanding on their optical properties.

### Characterizing the optical properties of AuNSs and AuNCs at the single particle level

**Figure 2:**
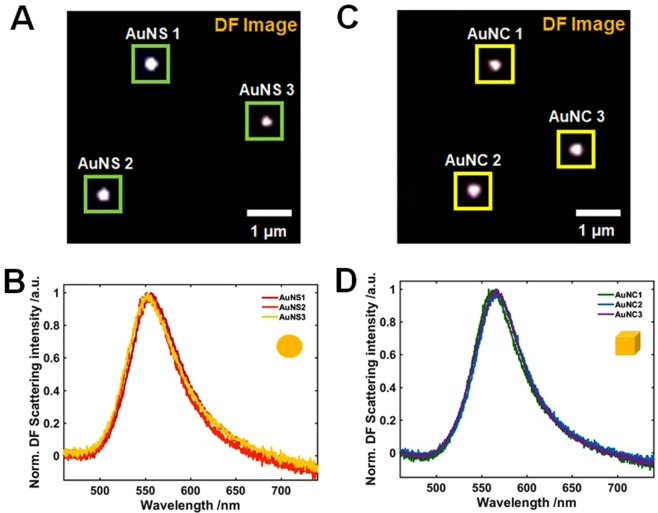
(**A**) Dark-field image of single AuNSs illuminated by white light. (**B**) Scattering spectra of the single AuNSs from the green square in (**A**). (**C**) Dark-field image of single AuNCs illuminated by white light. (**D**) Scattering spectra of the single AuNCs from the yellow square in (**C**).

Scattering-based DF microscopy and spectroscopy was used to characterize the shape-dependent optical properties of AuNSs and AuNCs with vertices at the single particle level^28^. The experimental setup for single particle DF microscopy and spectroscopy is shown in Fig. S2. The sample was prepared by drop casting aqueous solutions of the Au nanoparticles on a pre-cleaned glass slide for DF scattering measurements (Fig. S3). The prepared samples were then measured by illuminating with randomly-polarized white light tightly focused by a high NA oil condenser. Only the light scattered from the sample is collected by the objective lens under scattering-based DF microscopy and spectroscopy (Fig. S4). Figure 2A shows a DF scattering image of single AuNSs with an average size of 50.3 nm. In addition, the corresponding scattering spectra of three AuNSs, indicated by a green square in Fig. 2A, are presented in Fig. 2B. It can be observed that the single particle scattering spectra of AuNSs in water had a single broad LSPR peak at around 547 nm, which was further supported by the scattering spectra of more AuNSs (Fig. S5). Moreover, Fig. 2C presents the DF scattering image of single AuNCs with an average size of 51.1 nm, and single AuNCs with vertices also exhibited a single broad LSPR peak at around 567 nm (Figs 2D and S6). It is worth noting that AuNCs with vertices and AuNSs of similar size showed very similar single broad LSPR peaks in their scattering spectra. Furthermore, their LSPR peak shapes are not symmetrical.

### Effect of varying the medium dielectric constant on the LSPR wavelength shift

**Figure 3:**
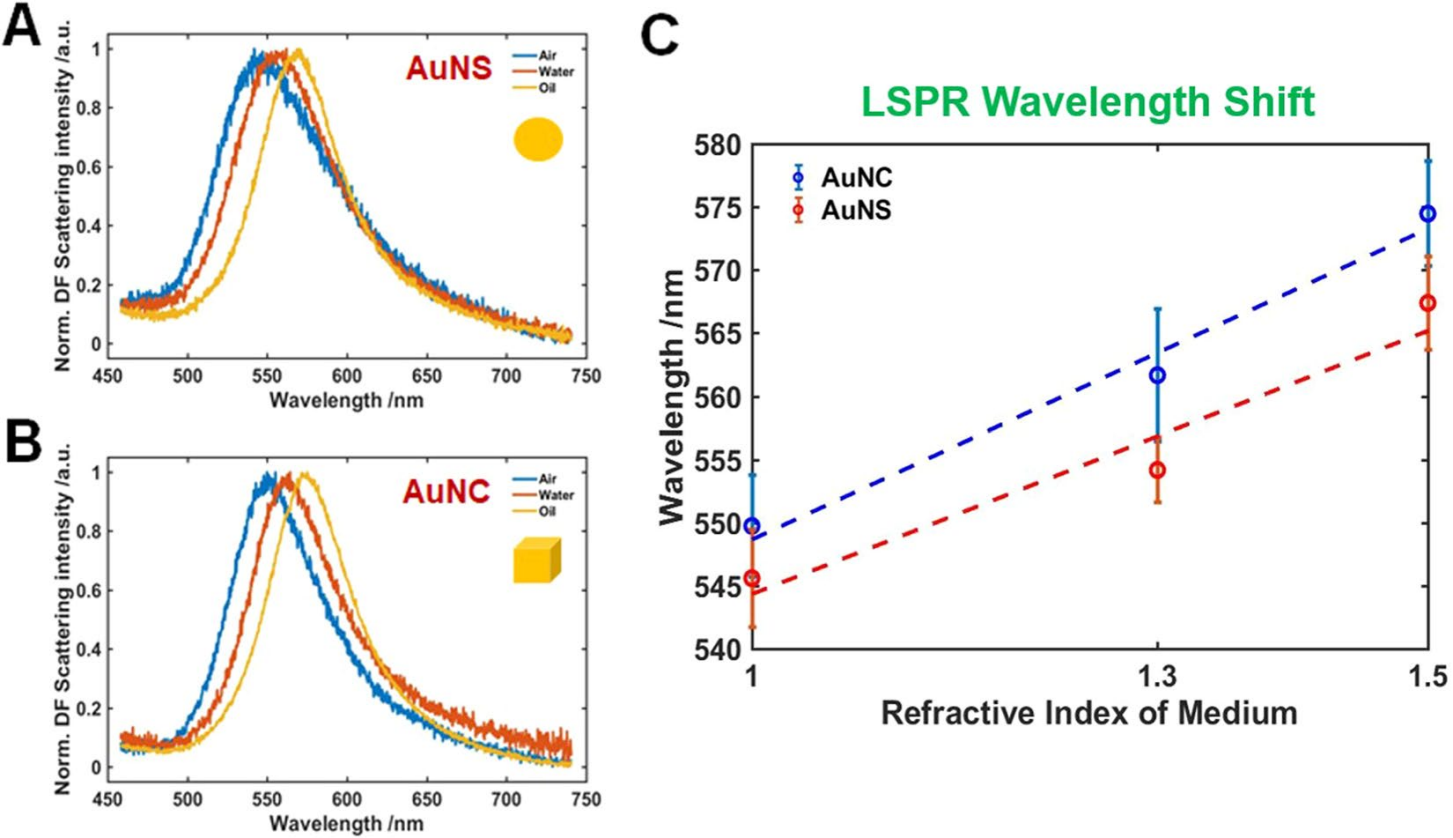
Change in the LSPR scattering spectra of single AuNS (**A**) and AuNC (**B**) in the different local RI media: air, water, oil. The scattering spectra represent average measurements as a demonstration of the LSPR peak shift with increasing the local RI from air to oil. (**C**) LSPR wavelength shifts for AuNS (red) and AuNC (blue) as a function of the local RI of medium.

To better understand the shape- and environment-dependent characteristic optical properties, the effect of changing the surrounding medium RI on the LSPR wavelength was further investigated. Therefore, the scattering spectra of single AuNSs and AuNCs were obtained in three different RI environments: air, water, and oil. Figure 3A presents the single particle scattering spectra of an AuNS fixed on a glass slide and surrounded by air, water, or oil. The LSPR spectrum was then fitted to a Lorentzian function to obtain the values of LSPR wavelength and linewidth (Fig. S7). As seen in this Fig. S7, the scattering spectra of single AuNS and AuNC were well fitted with the Lorentzian function. Figure 3A,B demonstrate that the LSPR wavelengths of both AuNS and AuNC increased as the RI increased from air to oil, which is consistent with previous studies^9,27^. Fig. 3C shows a comparison of the LSPR wavelength shifts as a function of RI of surrounding medium for AuNSs (red-curve) and AuNCs with vertices (blue-curve). Single AuNCs with sharp vertices showed a higher LSPR wavelength shift and RI sensitivity than spherical AuNSs of similar size. This indicates that single AuNCs with vertices could provide a higher RI sensitivity in the development of conventional LSPR sensors.

### Shape-dependent refractive index sensitivity of homogeneous LSPR inflection points

**Figure 4:**
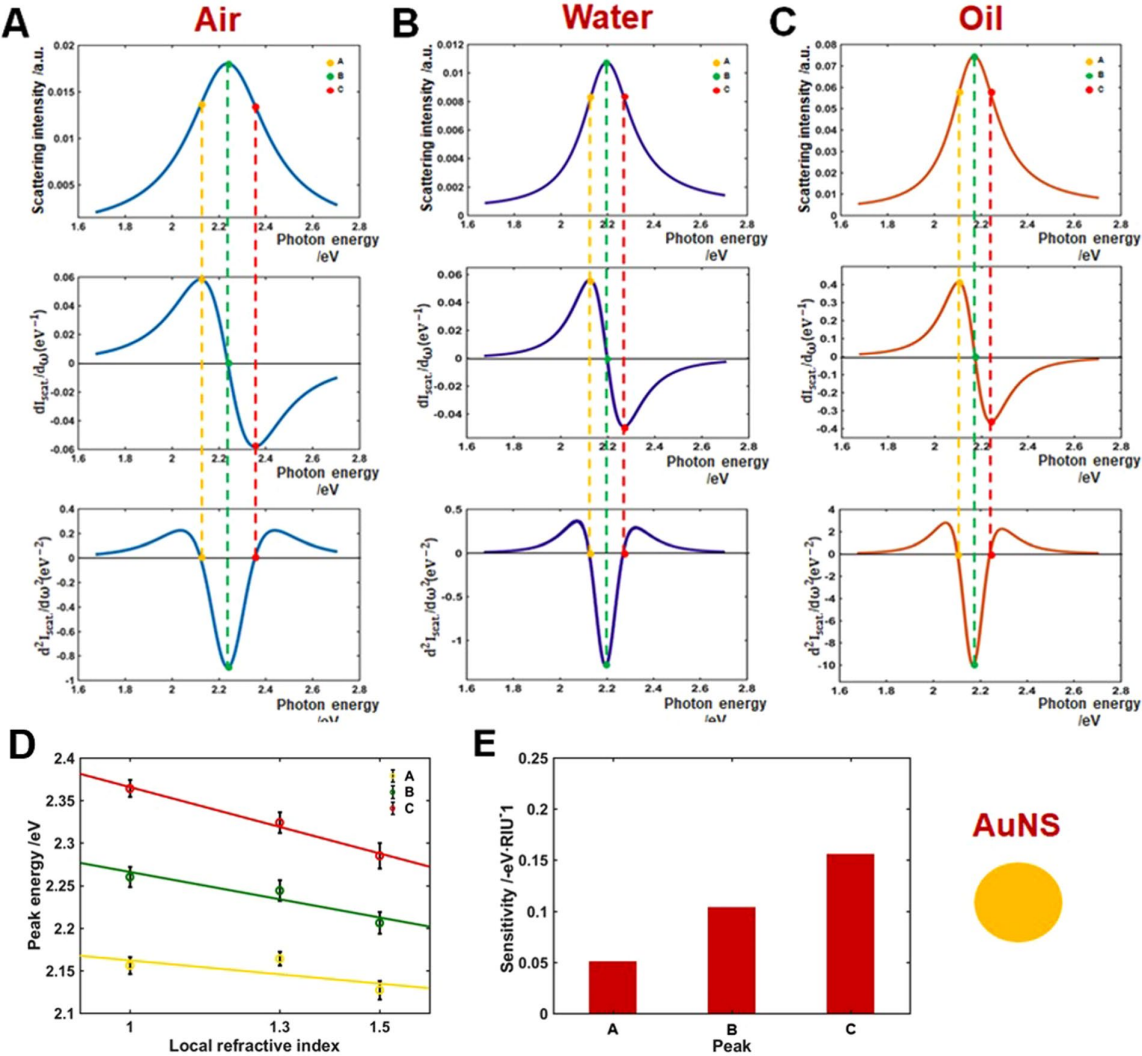
Inflection point method for single particle LSPR scattering sensing with AuNSs in the three local refractive indexes (air, water, and oil). (**A**–**C**) LSPR scattering efficiencies (first row), and its first (second row), and second (third row) order derivatives. (**D**) Peak energy plotted against the three local RI for points A, B, and C. (**E**) Sensitivity of local RI media on peak shifts A, B and C.

Homogeneous LSPR inflection points (IFs, e.g., the long wavelength side) have been reported to have a higher RI sensitivity than the LSPR wavelength maximum peak in single Au bipyramids with sharp tips^27^. However, it is necessary to deepen our understanding on the RI sensitivity of homogeneous LSPR IFs of various Au nanoparticles with different shapes, such as multiple sharp branches, vertices, etc. We therefore investigated the shape-dependent RI sensitivity of LSPR IFs in the homogeneous scattering spectra of both AuNSs and AuNCs. The first and second derivatives of the scattering spectra taken from DF experiments were obtained using a convenient method based on the Lorentzian fitting curve function^27^. The first, second, and third rows in Fig. 4A–C show the scattering spectra of single AuNS and the corresponding first and second order derivatives, respectively. Each column corresponds to one of the three local RI media used (air, water, and oil). The maxima of the LSPR scattering peak, indicated as B, are located at 2.236, 2.194, and 2.170 eV for the three-different RI environments (air, water, and oil). Moreover, the local maxima and minima of the first order derivatives, A and C, are located at 2.137 and 2.335, 2.126 and 2.270, and 2.104 eV and 2.241 eV for air, water, and oil, respectively. Consequently, A and C represent the two LSPR IFs, yielding the zero values of the second order derivatives of the LSPR scattering spectra (third row). It is worth noting that the LSPR IFs coincide with the local maxima/minima of the first order derivatives and appear at the same points of A and C on the axis corresponding to photon energy for the three different RI media. As observed in the first order derivative, B appears to be the critical point of the LSPR scattering spectra of AuNS, which indicates the zero values of the first order derivative spectra.

As shown in Fig. 4, the characteristic shapes of the LSPR scattering spectra of single AuNSs in the first and second order derivatives are consistent with a previous report on LSPR IFs obtained from the extinction spectra of gold nanoparticles measured at the ensemble level^25^. Furthermore, the zero values of the first order derivatives axis are exactly at the point B (LSPR peak maxima), which is the point of symmetry for the three local RI media. When analyzing the curvatures, it was found that the LSPR scattering curves and second order derivatives are even functions and symmetrical to the axis of intensity, while the first order derivative curves are odd functions and are symmetrical to the axis of photon energy.

As shown in the Supplementary Information (Tables S1–S3), the LSPR scattering spectra of 10 more AuNSs for each local RI environment were obtained and analyzed to confirm the reproducibility and consistency with the experimental results (Fig. 4). The experimental data was consistent for all the AuNSs evaluated, yielding LSPR peak maxima (B) of 2.228 (±0.043), 2.185 (±0.034), and 2.168 (±0.019) eV for the local air, water, and oil media. The LSPR IFs values, (A) and (C), were 2.127 (±0.029) and 2.329 (±0.057), 2.109 (±0.032) and 2.267 (±0.038), and 2.099 (±0.019) and 2.242 (±0.020) eV, respectively. Furthermore, considering the regime relevant to sensing properties, in which the peak energies should be approximately linear functions of the local RI media^29^, the linearity of the A, B, and C peak energies was examined for air, water, and oil. Figure 4D shows the plots of the energy peaks A, B, and C against local air, water, and oil media with corresponding RI values of 1.00, 1.33, and 1.52. As seen in the Fig., the relationship between the peak energies at A, B, and C and the local RI media was linear. The slopes, determined form a fitting function, were 0.064 eV·RIU^−1^ (R^2^ = 0.9398) for peak A, 0.133 eV·RIU^−1^ (R^2^ = 0.9983) for B, and 0.190 eV·RIU^−1^ (R^2^ = 0.9895) for C. It should be noted that the inflection point C exhibited the highest sensitivity with respect to A and the LSPR peak maxima (B), as shown in Fig. 4E. Further details are provided in the Supplementary Information (Tables S1–S3 and S7). Interestingly, the local RI sensitivity at inflection point C was improved by 5.00% compared to that at the LSPR peak maximum (B). This is consistent with previous reports using gold ensembles and single Au bipyramids for the utilization of LSPR IFs to enhance RI sensitivity^23,25,27^.

Next, to better understand the shape-dependent RI sensitivity at the LSPR IFs, DF microscopy and spectroscopy experiments were performed for AuNCs with vertices. The RI sensitivity of LSPR IFs of AuNSs was compared with that of AuNCs with vertices. Both AuNSs and AuNCs of similar size showed a single broad LSPR peak at similar LSPR wavelengths; therefore, this investigation focuses on how the shape of the nanoparticles (e.g., vertices, edges, etc.) affects the RI sensitivity at LSPR IFs at the single particle level.

**Figure 5:**
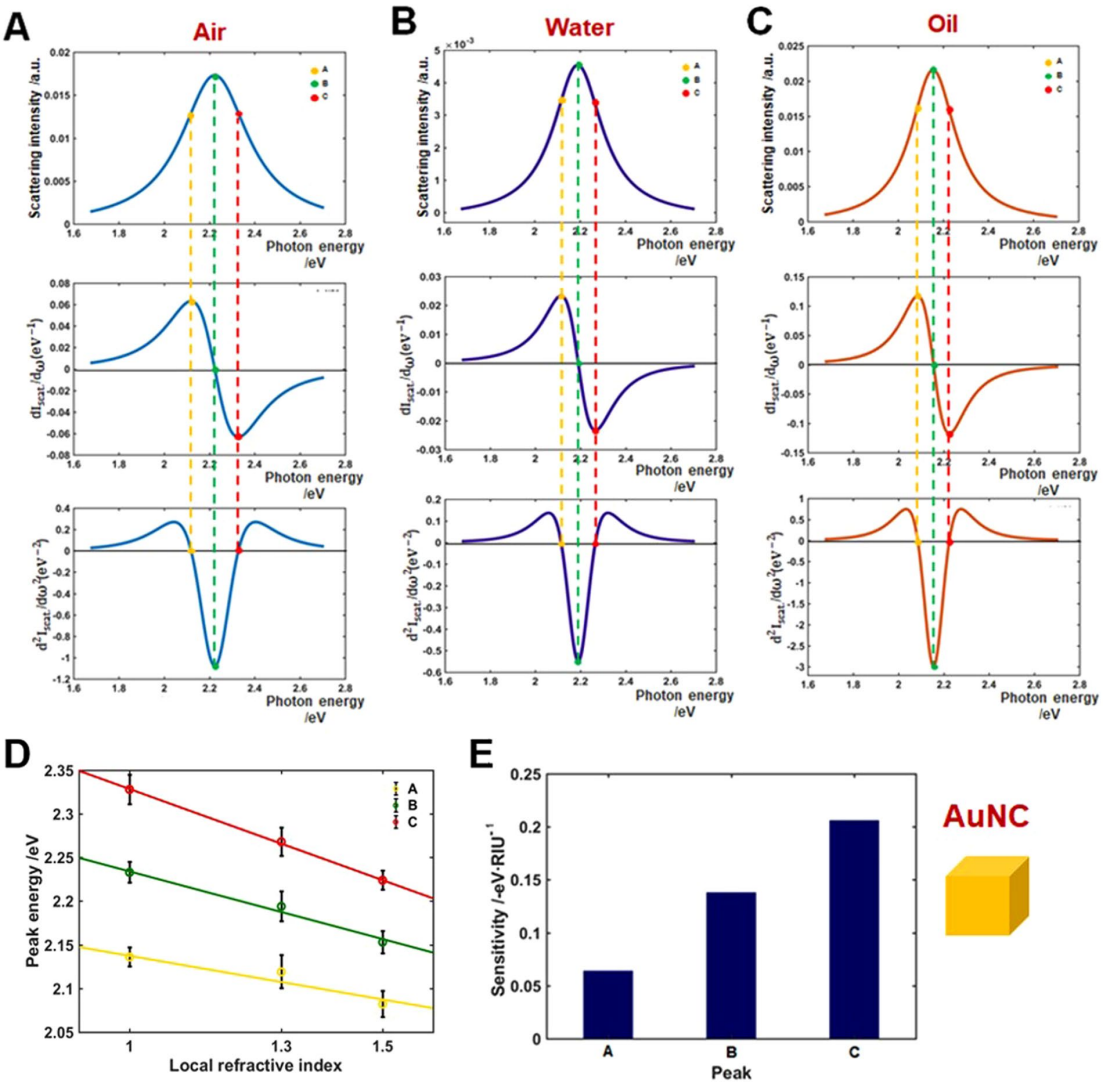
Inflection point method for single particle LSPR scattering sensing with AuNCs in the three local refractive indexes (air, water, and oil). (**A**–**C**) LSPR scattering efficiencies (first row), and its first (second row), and second (third row) order derivatives. (**D**) Peak energy plotted against the three local RI for points A, B, and C. (**E**) Sensitivity of local RI media on peak shifts A, B and C.

Similar to the analysis method used for AuNSs in Fig. 4, the first and second derivatives of the experimental LSPR scattering spectra of AuNCs with vertices were obtained. The first, second, and third rows in Fig. 5A–C show the scattering spectra of single AuNCs and the corresponding first and second order derivatives, respectively. The maxima of the LSPR scattering peak in the three local RI media, B, are located at 2.225, 2.190, and 2.155 eV for the three local environments (air, water, and oil). The local maxima and minima of the first order derivatives flanking the LSPR peak maxima (B), represented by A/C, are at 2.119/2.328, 2.114/2.265 and 2.085/2.225 eV for air, water, and oil, respectively. Consequently, A and C represent the two LSPR IFs of AuNCs, yielding the zero values of the second order derivatives of the LSPR scattering spectra (third row).

Measurement of the LSPR (B, maximum) scattering spectra in multiples of 10 for each local RI index provided the same results, with values of 2.233 (±0.012), 2.194 (±0.017) and 2.153 (±0.013) eV for local air, water, and oil. Similarly, the LSPR IFs, A and C, were 2.136 (±0.011) and 2.328 (±0.017), 2.119 (±0.019) and 2.268 (±0.016), and 2.082 (±0.015) and 2.224 (±0.011) eV, respectively. The peak energy A, B, and C was plotted *vs*. local air, water, and oil RI media. As presented in Fig. 5D, the peak energies at A, B, and C showed a linear relation with the three different local RI media. The use of a fitting function allowed to determine the slopes: 0.064 eV·RIU^−1^ (R^2^ = 0.9685) for peak A, 0.138 eV·RIU^−1^ (R^2^ = 0.9868) for B, and 0.206 eV·RIU^−1^ (R^2^ = 0.9998) for C. Similar to the experimental result of AuNSs, the inflection point C exhibited the highest sensitivity with respect to the IF A and the LSPR peaks maxima (B) as shown in Fig. 5E (Tables S4–S6, and S8 for full details). Interestingly, the local RI sensitivity at inflection point C was improved by 5.10% with respect to the LSPR peak maximum (B). This result is consistent with that of AuNSs (Fig. 4). Therefore, the LSPR IF C at the longer wavelength side showed higher RI sensitivity than the LSPR peak maximum (B) for both AuNSs and AuNCs. Furthermore, AuNCs with edges and vertices showed higher RI sensitivity than AuNSs of similar size at the position of LSPR IF C (Fig. S8).

## Conclusions

In summary, we demonstrated the significance of tracking the curvature shapes through homogeneous LSPR IFs near the resonance energy in various local RIs (air, water, oil), rather than tracking their counterpart LSPR maximum peak shifts, for both AuNSs and AuNCs of similar size. The homogeneous LSPR scattering IFs of single gold nanoparticles (AuNSs, AuNCs) with a single resonant mode showed an enhanced RI sensitivity in various local RI environments. Furthermore, we found that single AuNCs with sharp vertices and edges showed higher RI sensitivity at homogeneous LSPR IFs than single AuNSs, with no edges, of similar size. Therefore, this study provides a deep insight into shape-dependent RI sensitivity of homogeneous LSPR IFs in single Au nanoparticles having a single resonant mode using DF single particle spectroscopy. Moreover, we showed that tracking the curvature changes in the LSPR scattering spectra of single AuNCs with vertices may be effectively employed in LSPR-based RI sensing studies.

## Methods

### Materials

Cetyltrimethylammonium bromide (CTAB)-stabilized gold nanospheres (AuNSs) and gold nanocubes (AuNCs) with an average size of 50 nm were purchased from Nanopartz (Loveland, CO, USA). Immersion oil was purchased from Sigma-Aldrich (St. Louis, MO, USA).

### Characterization of gold nanospheres and gold nanocubes with vertices

The structural characterization of AuNSs and AuNCs was conducted by scanning electron microscopy (SEM, JSM-6500, JEOL, Japan) to assess the shapes and sizes. Furthermore, the LSPR absorption spectra of the AuNSs and AuNCs dispersed in water were measured using a Varian Carry 300 UV-Vis spectrophotometer (Agilent, USA).

### Sample preparation for single particle study

The preparation of the samples was simple. First, the colloid solution was diluted with distilled water to lower the concentration. The diluted solution was sonicated for 10 min at room temperature and was then dropped on a washed slide glass and covered with a 22 mm × 22 mm No. 1.5 cover glass (Corning, NY). To achieve the conditions of air as surrounding medium, the aqueous solution on the slide glass was dried after placing the cover glass. When using the oil as surrounding medium, the same procedure was followed and then, after drying the aqueous solution, the immersion oil was added. The concentration of Au nanoparticles deposited on the glass slide was adjusted to approximately 1 μm^−2^ to facilitate the measurement of a single particle without inter-particle LSPR coupling.

### Single particle microscopy and spectroscopy

We performed scattering-based dark-field (DF) microscopy using an inverted microscope (ECLIPSE Ti-U, NIKON, Japan). In the DF mode, we used a Nikon Plan Fluor oil iris objective (100×) with an adjustable numerical aperture (NA, 0.5–1.3) and a Nikon DF condenser for DF imaging. To obtain DF scattering images with high quality, we used an Andor EMCCD camera (iXon Ultra 897, UK). We analyzed the collected DF images with the Image J software. Furthermore, single particle spectra of AuNSs and AuNCs were taken by using an Andor spectrometer (SHAMROCK303i, SR-303I-A, UK) equipped with an Andor CCD camera (Newton DU920P-OE, UK). We collected the scattered light from AuNPs by an objective lens and sent to the entrance of the spectrometer for taking a spectrum. The scattered light was then dispersed by a grating (300 l/mm) inside the spectrometer, and detected by the Andor CCD camera (Newton DU920P-OE, UK). We obtained a background spectrum at an area without nanoparticles. Finally, Matlab programs specially designed for this study were used to perform data analysis and to obtain single particle spectra.

## Supplementary information

Supplementary Information
