# Supplementary material for: Shape Effect on the Refractive Index Sensitivity at Localized Surface Plasmon Resonance Inflection Points of Single Gold Nanocubes with Vertices
Source: Sci Rep. 2019 Sep 20;9:13635. doi: 10.1038/s41598-019-50032-3 (PMC6754453; doi:10.1038/s41598-019-50032-3)
Supplement: Supplementary file 1 — Supplementary Information [file 41598_2019_50032_MOESM1_ESM.pdf]

Supplementary Information

Shape Effect on the Refractive Index Sensitivity at Localized Surface Plasmon Resonance Inflection Points of Single Gold Nanocubes with Vertices

Hui Bin Jeon,[1] Philippe Vuka Tsalu,[1] and Ji Won Ha [1*]

[1]Advanced Nano-Bio-Imaging and Spectroscopy Laboratory, Department of Chemistry, University of Ulsan, 93 Daehak-Ro, Nam-Gu, Ulsan 44610, South Korea

[*]To whom correspondence should be addressed.

J. W. Ha
Phone: +82-52-712-8012
Fax: +82-52-712-8003
E-mail: jwha77@ulsan.ac.kr

This document contains supplementary tables (Table S1 to S8) and figures (Fig. S1 to S8).

Supplementary Tables

Single AuNSs	1	2	3	4	5	6	7	8	9	10	Ave	Std
Inflection A (eV)	2.156	2.154	2.173	2.167	2.161	2.135	2.153	2.154	2.144	2.160	2.156	0.010
LSPR B (eV)	2.258	2.264	2.271	2.272	2.260	2.245	2.261	2.249	2.254	2.263	2.260	0.008
Inflection C (eV)	2.360	2.372	2.370	2.387	2.358	2.353	2.367	2.345	2.363	2.367	2.364	0.011

Table S1. Inflection points and LSPR peak locations on the curvatures of single AuNSs in refractive index of air

Single AuNSs	1	2	3	4	5	6	7	8	9	10	Ave	Std
Inflection A (eV)	2.161	2.173	2.186	2.174	2.157	2.152	2.145	2.176	2.156	2.158	2.164	0.012
LSPR B (eV)	2.244	2.246	2.257	2.260	2.250	2.241	2.219	2.254	2.241	2.230	2.244	0.012
Inflection C (eV)	2.320	2.320	2.329	2.342	2.339	2.325	2.299	2.335	2.324	2.306	2.324	0.013

Table S2. Inflection points and LSPR peak locations on the curvatures of single AuNSs in refractive index of water

Single AuNSs	1	2	3	4	5	6	7	8	9	10	Ave	Std
Inflection A (eV)	2.127	2.133	2.130	2.120	2.110	2.140	2.126	2.111	2.139	2.139	2.127	0.010
LSPR B (eV)	2.205	2.219	2.206	2.207	2.186	2.218	2.201	2.185	2.220	2.216	2.206	0.012
Inflection C (eV)	2.283	2.307	2.281	2.294	2.262	2.295	2.278	2.258	2.301	2.293	2.285	0.015

Table S3. Inflection points and LSPR peak locations on the curvatures of single AuNSs in refractive index of oil

Single AuNCs	1	2	3	4	5	6	7	8	9	10	Ave	Std
Inflection A (eV)	2.143	2.141	2.139	2.137	2.119	2.152	2.129	2.132	2.151	2.119	2.136	0.011
LSPR B (eV)	2.239	2.236	2.244	2.248	2.225	2.237	2.216	2.219	2.245	2.216	2.233	0.012
Inflection C (eV)	2.333	2.330	2.349	2.357	2.328	2.320	2.304	2.306	2.337	2.312	2.328	0.017

Table S4. Inflection points and LSPR peak locations on the curvatures of single AuNCs in refractive index of air

Single AuNCs	1	2	3	4	5	6	7	8	9	10	Ave	Std
Inflection A (eV)	2.115	2.133	2.115	2.103	2.114	2.109	2.144	2.129	2.080	2.143	2.119	0.019
LSPR B (eV)	2.190	2.205	2.190	2.178	2.190	2.185	2.216	2.206	2.158	2.217	2.194	0.017
Inflection C (eV)	2.264	2.277	2.264	2.252	2.265	2.260	2.288	2.284	2.236	2.291	2.268	0.016

Table S5. Inflection points and LSPR peak locations on the curvatures of single AuNCs in refractive index of water

Single AuNCs	1	2	3	4	5	6	7	8	9	10	Ave	Std
Inflection A (eV)	2.095	2.077	2.051	2.093	2.089	2.096	2.087	2.085	2.093	2.058	2.082	0.015
LSPR B (eV)	2.165	2.146	2.126	2.163	2.159	2.166	2.157	2.155	2.161	2.135	2.153	0.013
Inflection C (eV)	2.232	2.214	2.200	2.232	2.230	2.235	2.226	2.225	2.231	2.212	2.224	0.011

Table S6. Inflection points and LSPR peak locations on the curvatures of single AuNCs in refractive index of oil

Single AuNSs	1	2	3	4	5	6	7	8	9	10	Ave	Std
Slope at Inflection A ($eV \cdot RIU^{-1}$)	0.052	0.034	0.076	0.085	0.095	0.014	0.052	0.073	0.005	0.039	0.052	0.028
Slope at LSPR B ($eV \cdot RIU^{-1}$)	0.101	0.088	0.123	0.123	0.139	0.051	0.122	0.117	0.066	0.095	0.102	0.026
Slope at Inflection C ($eV \cdot RIU^{-1}$)	0.152	0.133	0.175	0.183	0.182	0.114	0.182	0.163	0.124	0.152	0.156	0.024

Table S7. Local refractive index Sensitivities of single AuNSs at each point (first inflection point A, LSPR peak B, second inflection point C)

Single AuNCs	1	2	3	4	5	6	7	8	9	10	Ave	Std
Slope at Inflection A ($eV \cdot RIU^{-1}$)	0.096	0.120	0.168	0.090	0.057	0.115	0.073	0.087	0.126	0.106	0.104	0.029
Slope at LSPR B ($eV \cdot RIU^{-1}$)	0.149	0.174	0.232	0.175	0.131	0.145	0.109	0.121	0.178	0.149	0.156	0.033
Slope at Inflection C ($eV \cdot RIU^{-1}$)	0.204	0.228	0.297	0.258	0.197	0.172	0.148	0.155	0.222	0.190	0.207	0.044

Table S8. Local refractive index Sensitivities of single AuNCs at each point (first inflection point A, LSPR peak B, second inflection point C)

Supplementary Figures

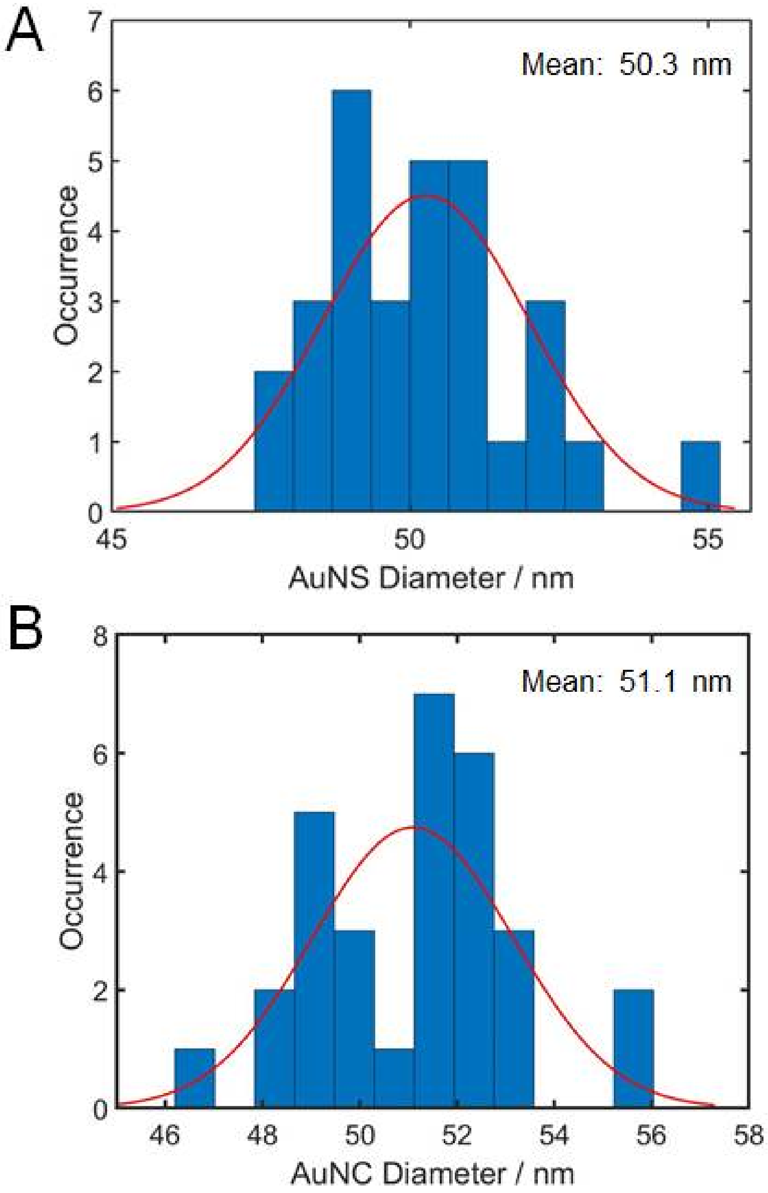

Figure S1. (A) Histogram to show the size distribution of AuNSs. **(B)** Histogram to show the size distribution of AuNCs with vertices used in this study.

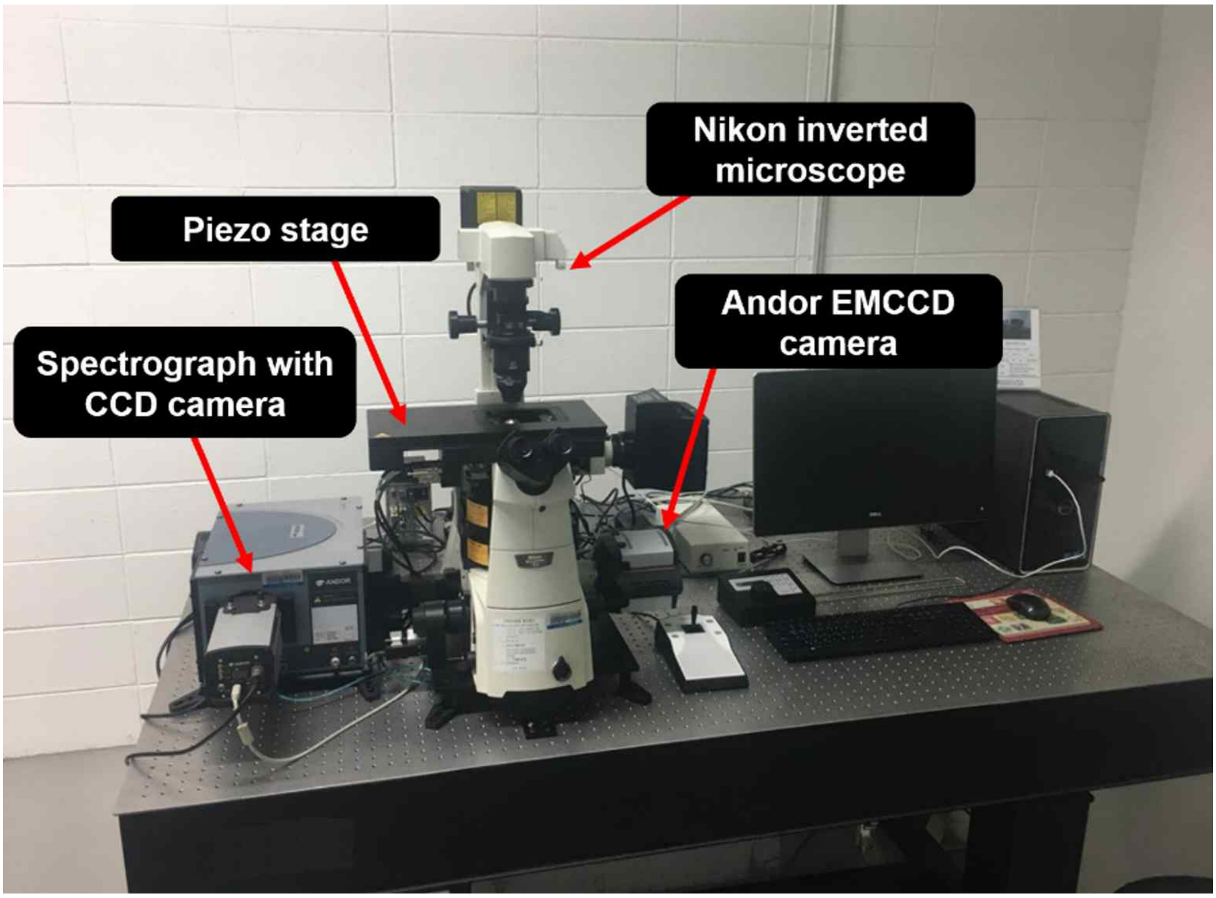

Figure S2. A photograph to show the experimental setup for single particle DF microscopy and spectroscopy.

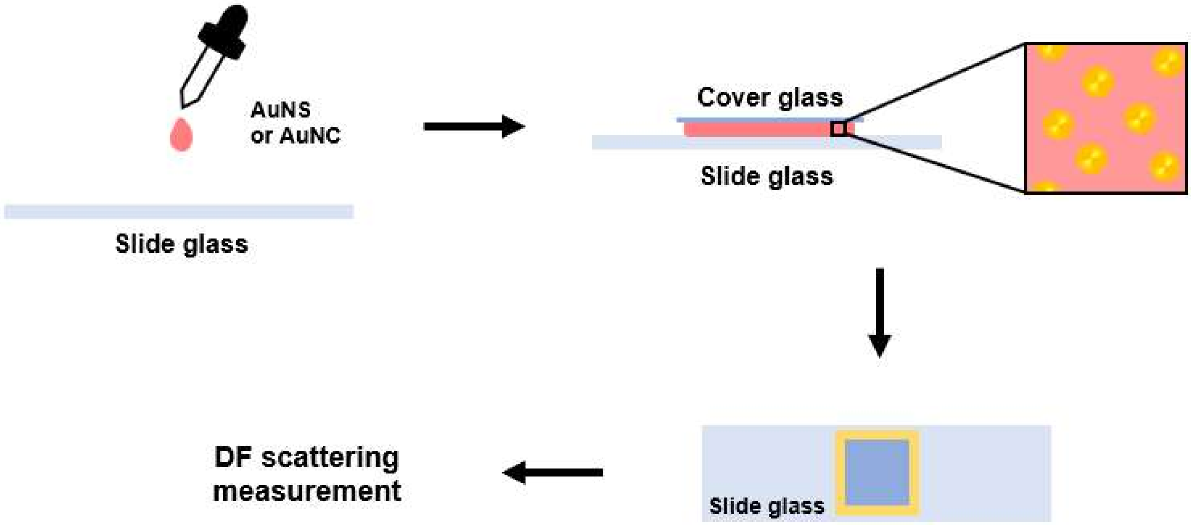

Figure S3. Schematic depicting the preparation of the samples for DF scattering measurement.

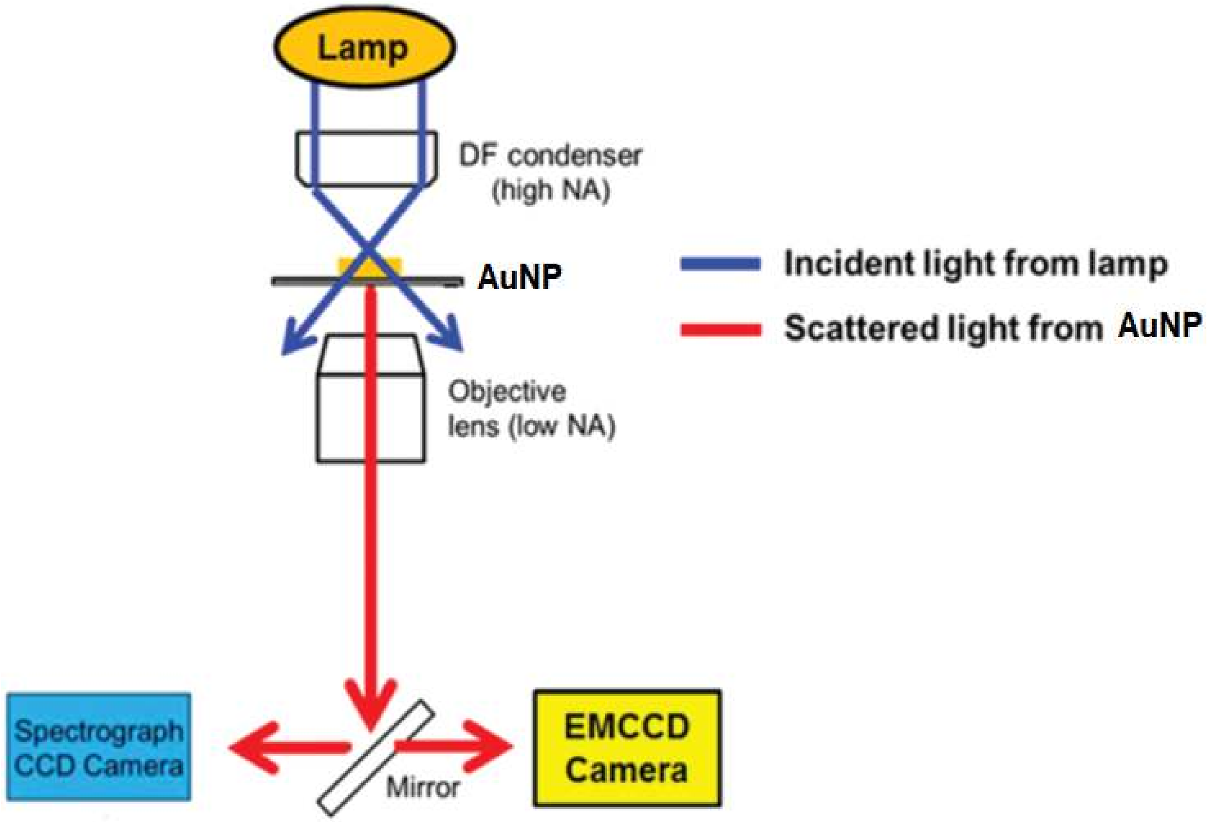

Figure S4. Schematic depicting the working principle of scattering-based DF microscopy and spectroscopy.

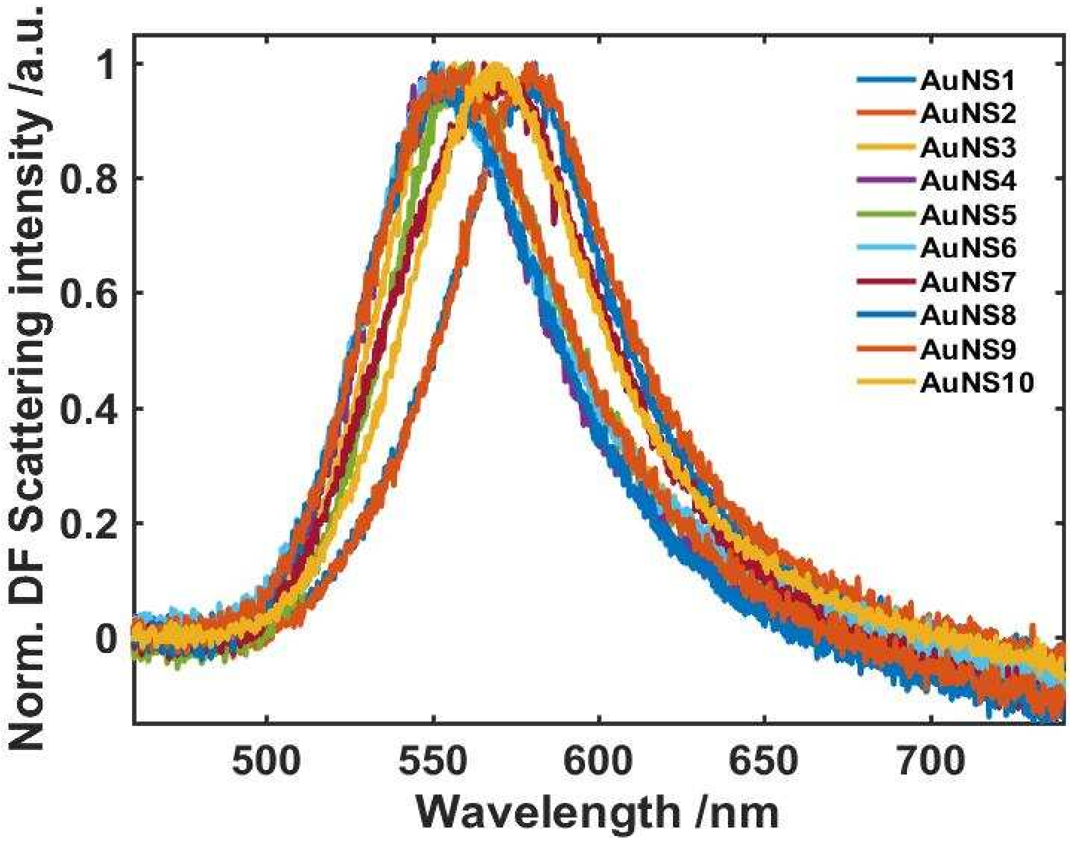

Figure S5. Single particle scattering spectra of 10 more AuNSs.

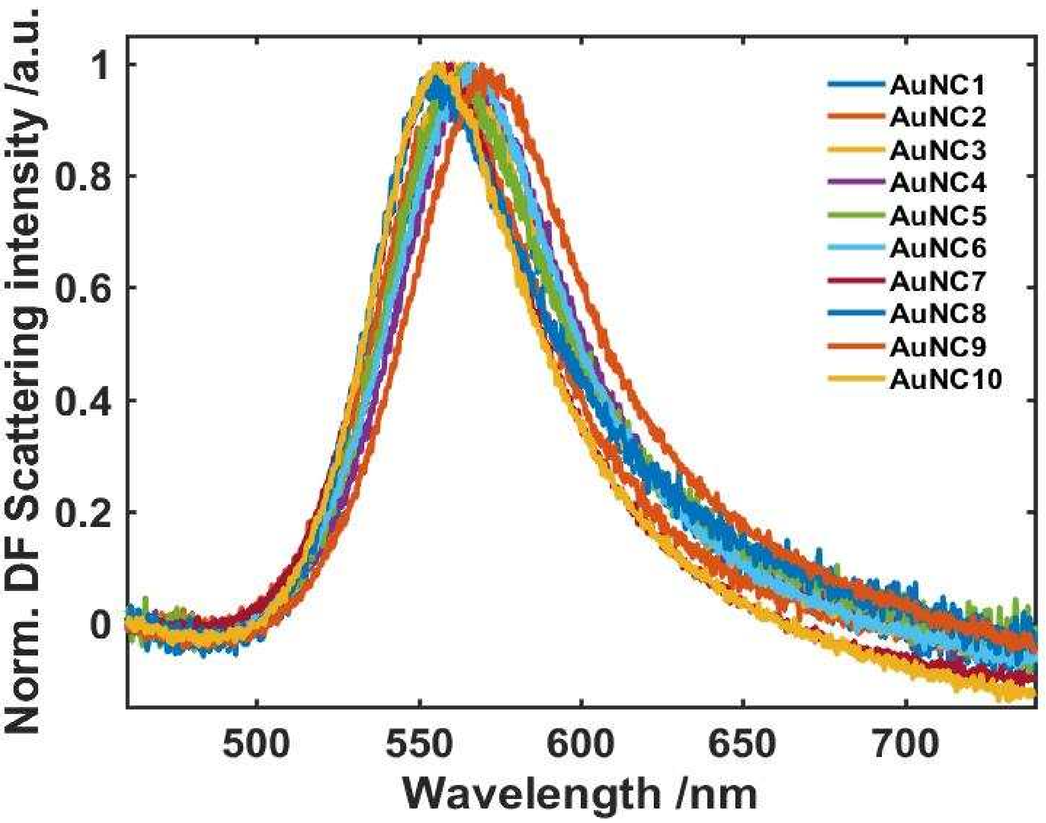

Figure S6. Single particle scattering spectra of 10 more AuNCs with vertices.

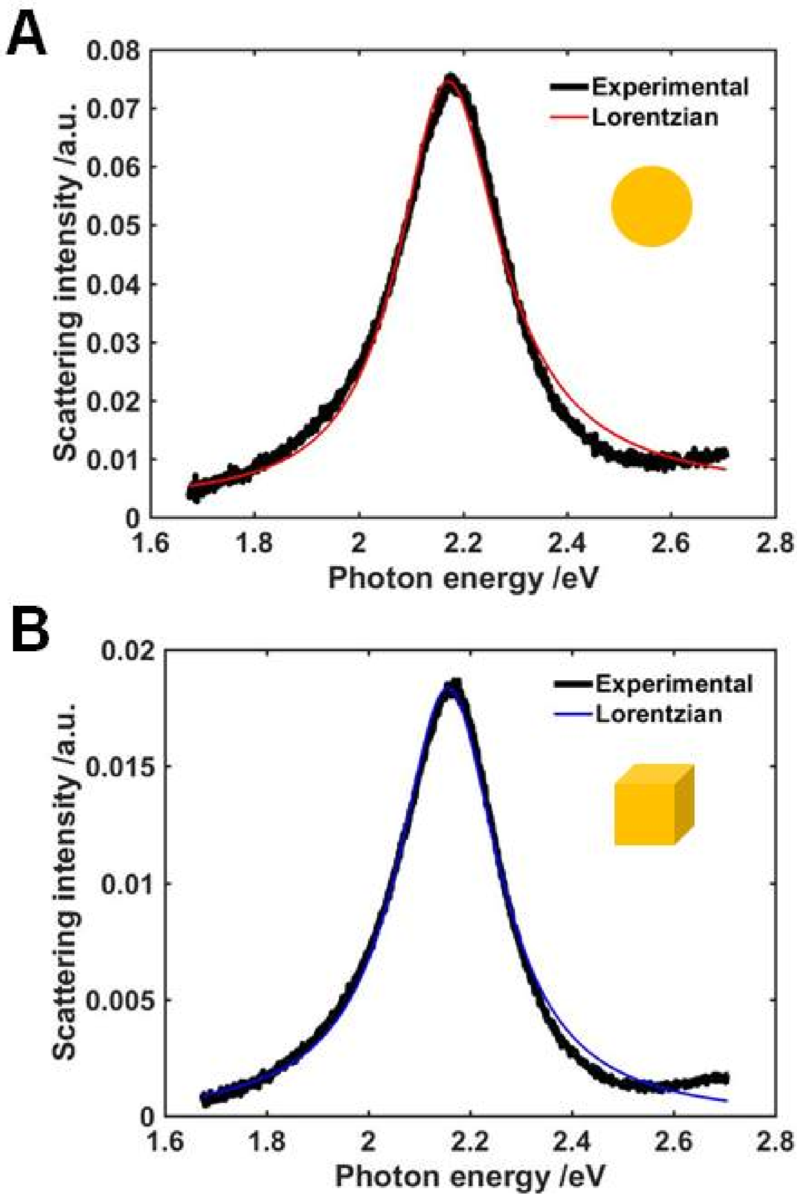

Figure S7. Lorentzian fitting to experimental scattering spectra of **(A)** AuNS and **(B)** AuNC with vertices. The experimental scattering spectra were well fitted to a Lorentzian function for both AuNS and AuNC with a single resonant mode.

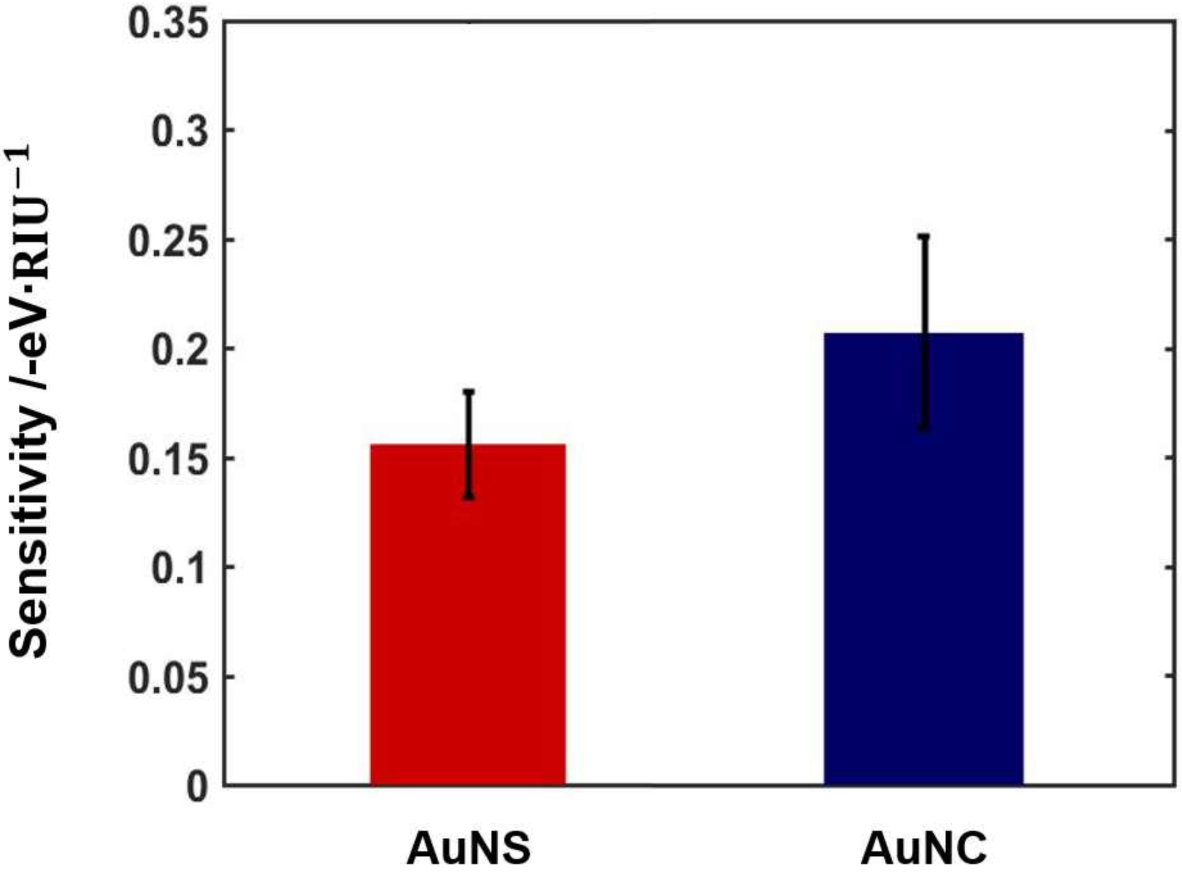

Figure S8. Comparison of RI sensitivity of the homogeneous LSPR IF at the long wavelength side for AuNSs (left, red-color) and AuNCs with vertices (right, blue-color).
